# Supraphysiological effects of pancreatic polypeptide on gastric motor function and nutrient tolerance in humans

**DOI:** 10.14814/phy2.15002

**Published:** 2021-08-26

**Authors:** Wout Verbeure, Alessandra Rotondo, Pieter Janssen, Florencia Carbone, Jan Tack

**Affiliations:** ^1^ Translational Research Center for Gastrointestinal Disorders KULeuven Belgium

**Keywords:** food tolerance, gastric accommodation, gastric emptying, pancreatic polypeptide

## Abstract

Pancreatic polypeptide (PP) is known to affect food intake. In this exploratory study, we set out to investigate its supraphysiological effect on food tolerance, gastric accommodation, and emptying. In 12 healthy volunteers, 0, 3, or 10 pmol*kg^−1^*min^−1^ PP was administered intravenously (PP0, PP3 or PP10). Thirty minutes thereafter, nutrient drink infusion (60 ml*min^−1^) through a nasogastric feeding tube was started until maximum satiation. Gastric accommodation was assessed by measuring the intragastric pressure (IGP; nasogastric manometry). In a separate test, the effect of PP0 or PP10 on gastric emptying was tested in 10 healthy volunteers and assessed using the ^13^C breath test. Results are presented as mean ± SEM, and *p* < 0.05 was considered significant. For the IGP test, PP increased ingested nutrient volume: 886 ± 93, 1059 ± 124, and 1025 ± 125 ml for PP0, PP3, and PP10, respectively (*p* = 0.048). In all groups, Nadir IGP values were reached upon food intake (transformed values: 1.5 ± 0.2, 1.7 ± 0.3, and 1.6 ± 0.3 mmHg for PP0, PP3, and PP10, respectively; NS) to return to baseline thereafter. For the gastric emptying study, volunteers ingested a similar nutrient volume: 802 ± 119 and 1089 ± 128 ml (*p* = 0.016), and gastric half‐emptying time was 281 ± 52 and 249 ± 37 min for PP0 and PP10, respectively (NS). No significant correlation between tolerated nutrient volume and IGP drop (R² < 0.01; *p* = 0.88 for PP0 vs. PP3 and R² =0.07; *p* = 0.40 for PP0 vs. PP10, respectively) or gastric half‐emptying time (R² = 0.12; *p* = 0.32) was found. A supraphysiological PP dose enhances food tolerance; however, this effect is not mediated through gastric motility.

**Clinical Trial registry number:**

NCT03854708 is obtained from clinicaltrials.gov.

## Graphical abstract

In this exploratory study, we set out to investigate the supraphysiological effect of pancreatic polypeptide on food tolerance, gastric accommodation, and emptying. Interestingly, this dose enhances the tolerated amount of an infused meal, which is contradictory with literature using physiological dosages. The effect was not mediated through gastric motility.

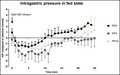

## 1 INTRODUCTION

Pancreatic polypeptide (PP) is a 36‐amino acid peptide released into the circulation from the endocrine F cells of the peripheral pancreatic islets after a meal and/or exercise. PP release occurs at a low rate in the fasted state but it is strongly increased during the meal digestion (Kojima et al., 2007). PP secretion is biphasic and depends on the amount of food ingested but is independent from the nutrient composition of the meal (Kojima et al., 2007). PP shows consistent structural homology with neuropeptide Y and peptide YY (Ekblad & Sundler, 2002; Tatemoto et al., 1982) and exerts its effect by binding the G‐protein‐coupled Y‐receptors whose five different subtypes Y1–Y5 are expressed in mammals (Larhammar, 1996). PP binds with greatest affinity the Y4 receptor, which mRNA is expressed in regions of the dorsal vagal complex, the area postrema (AP), and nucleus tractus solitarius (NTS) (Larsen & Kristensen, 1997; Parker & Herzog, 1999), modulating the vago‐vagal reflex pathways and leading to changes in gastrointestinal functions such as pancreatic exocrine secretion, gallbladder contraction, and gastric motility (Kojima et al., 2007). Moreover, PP also seems to be involved in the modulation of food intake. It has been reported that the peripheral administration of PP exerts anorexigenic effects in humans, causing suppressed food intake (Batterham et al., 2003). On the other hand, studies in rodents and dogs have shown an increase of food intake after central administration of PP (Clark et al., 1984; Inui et al., 1991). Moreover, altered PP secretion has been reported in clinical syndromes associated with abnormal eating behavior in humans. Patients with Prader–Willi syndrome, a genetic form of obesity characterized by extreme hyperphagia, have a blunted PP response following a meal (Zipf et al., 1981). Blunted postprandial PP release has also been observed in individuals with morbid obesity (Lieverse et al., 1994), whereas subjects with anorexia nervosa show an exaggerated postprandial PP secretion (Fujimoto et al., 1997). Exogenous administration of PP was followed by decreased food intake in both Prader–Willi syndrome and healthy individuals (Batterham et al., 2003; Berntson et al., 1993). These observations indicate possible involvement of PP in the regulation of food intake and potentially also in the etiology of disorders of food intake such as obesity and anorexia nervosa.

The mechanism by which PP affects feeding behavior needs to be elucidated, but it is conceivable that at least part of this action could be mediated by the modulation of gastric motility (Kojima et al., 2007). However, studies on the effect of PP on gastric emptying rate are discordant, showing delay, enhancement, or lack of effect (Berntson et al., 1993; Kojima et al., 2007; Schmidt et al., 2005).

Besides gastric emptying, gastric accommodation is an important aspect of gastric motor function, and has been proposed to be a major determinant of food intake (Janssen, Vanden Berghe, et al., 2011). We have demonstrated that peripheral administration of PP in rats inhibits gastric accommodation and delays gastric emptying, probably through inhibition of nitric oxide release (Verschueren et al., 2014). However, so far, it remains unknown whether PP is also able to modulate gastric accommodation in humans and whether this could represent an additional mechanism by which PP can affect food intake and satiation feelings.

The aim of this study is to evaluate whether peripheral infusion of different supraphysiological doses of PP in healthy volunteers (HVs) can affect nutrient volume tolerance, hunger‐related sensations, gastric accommodation, and gastric emptying during intragastric nutrient drink (ND) infusion.

## 2 METHODS

### 2.1 Subjects

Twelve HVs were recruited for protocol 1 to study the effect of PP on gastric accommodation and 10 HVs for protocol 2 to study the effect on gastric emptying. Exclusion criteria comprised the presence of symptoms or a history of gastrointestinal diseases, any other significant disease or psychological disorder. None of the subjects was taking any medication. Informed consent was obtained from each participant and the study protocol was approved by the Ethics Committee of the Leuven University Hospital, Belgium and performed in full accordance with the declaration of Helsinki. The study, started in August 2010 and completed in March 2011, is published in clinicaltrials.gov with reference number NCT03854708.

### 2.2 Treatment and randomization

Both protocols were performed as a single blind, placebo‐controlled, crossover trial. In protocol 1, the effect of three different doses of PP: a 0.9% saline placebo (PP0), 3 pmol*kg^−1^*min^−1^ PP (PP3), and 10 pmol*kg^−1^*min^−1^ PP (PP10) on food tolerance was examined and whether it was mediated through gastric accommodation. For protocol 2, gastric emptying rate, food tolerance, and appetite‐related sensations (hunger, prospective food intake, and satiety) were evaluated in the PP0 and PP10 arm only. The PP was purchased from CS Bio (USA, California, Menlo Park) as a dry powder and was dissolved by the university hospital pharmacy in a 0.9% saline solution containing 5% albumin to the eventual doses of PP3 and PP10. The doses were intravenously (IV) infused 30 min before the meal until the end of the meal. For this purpose, a cannula was inserted into the subjects’ forearm vein.

Allocation happened in a randomized and counterbalanced order generated by a computer program (randomization.com). A minimum of 1 week was respected between consecutive visits.

### 2.3 Study protocols

#### 2.3.1 Protocol 1: Gastric accommodation and food tolerance

HVs came to the clinic after an overnight fast of at least 12 h. They refrained during this time from alcohol, tea, and coffee, and stopped smoking cigarettes at least 1 h before the start of the experiment. Volunteers were also asked to have a light bread supper as a last meal before the fasted period.

##### 2.3.1.1 Nutrient tolerance IGP measurement

Gastric accommodation during nutrient infusion was assessed by IGP monitoring as previously described (Janssen, Verschueren, et al., 2011). In brief, a 36‐channel high‐resolution solid‐state manometry probe (Manoscan 360; Sierra Scientific Instruments; Manoview analysis software v2.0.1) was positioned through the nose so that at least one sensor was positioned at the level of the lower esophageal sphincter (LES), while IGP was measured as the average pressure of five pressure channels that were clearly positioned below the LES or the pressure area influenced by the LES. A feeding tube (Flocare; Nutricia) was positioned in the stomach through the mouth. The tip of the infusion tube was positioned approximately 5 cm under the LES and its position was verified by fluoroscopy. The catheters were fixed to the subjects’ chin and the participants were comfortably positioned in a bed with the trunk upright. After a stabilization period of at least 15 min, the treatment was started. Thirty minutes thereafter, a liquid ND (Nutridrink®; 150 kcal, 6 g proteins, 18.4 g carbohydrates, 5.8 g lipids per 100 ml) was infused directly into the stomach at a constant speed (60 ml*min^−1^) determined by an automated system using a peristaltic pump. At 1‐min intervals, subjects were asked to score their satiation using a graphic rating scale that combines verbal descriptors on a scale graded from 0 to 5 (0, threshold; 5, maximal satiation). Both intragastric infusion and IV treatment were stopped as soon as the subject reached maximal satiation (score 5). Thereafter, the probe and the feeding tube were removed.

##### 2.3.1.2 Blood sample collection and human PP plasma level assay

After the catheter’ placement for PP infusion, an additional cannula was inserted into the other forearm in order to collect blood. Blood samples were taken 5 min before the PP IV infusion, 5 min before the intragastric ND infusion, at the end of the ND intragastric infusion, and 6 h after the meal. Samples were collected into 10‐ml tubes containing EDTA (BD Vacutainer® Plus plastic whole blood tube. Lavender BD Hemogard™ closure. Paper label. CE Additive: K_2_EDTA (spray dried) (100/sp, 1000/cs)) and aprotinin (50 kIU/ml, Millipore, Belgium). All samples were stored on ice until centrifugation at 4°C, after which plasma was separated and stored at −80°C until further analysis. PP content was assayed with an enzyme immunoassay kit (Pancreatic Polypeptide (Human) ‐ EIA Kit, Phoenix Europe GmbH, Germany).

### 2.4 Protocol 2: Gastric emptying and food tolerance

HVs came to the clinic after an overnight fast, and the same constraints were requested as in the previous protocol. A feeding tube was positioned into the stomach for the ND infusion and an intravenous catheter was placed into the subject's forearm for the infusion of the PP0 or PP10 treatment.

#### 2.4.1 Gastric emptying rate

Gastric emptying rate after PP0 and PP10 administration was quantified using the breath test (Braden et al., 1995). ^13^C‐labeled sodium octanoate (200 mg*L^−1^) was added to the ND (250 ml, 150 kcal per 100 ml), and the liquid meal was infused at a constant speed (60 ml*min^−1^) as discussed in the previous protocol. Volunteers scored their satiation on a scale and stopped as soon as the subject reached maximal satiation. Breath samples were collected in exetainers (Labco, UK), twice before and every 15 min after the meal until 6 h thereafter. The gastric half‐emptying time (T_1/2_) was determined with the ratio of the exhaled ^13^CO_2_/^12^CO_2_ by using mass spectrometry.

#### 2.4.2 Satiety, return of hunger, and prospective food intake

HVs scored their feeling of satiety, return of hunger, and prospective food intake using a VAS of 100 mm simultaneously with the collection of the breath samples for gastric emptying.

### 2.5 Data analysis and statistical test

#### 2.5.1 Intragastric pressure measurement

The original data were imported from the recording software to Microsoft Excel (Microsoft). In order to avoid influence from artefacts caused by coughing, sneezing, subject movement, or swallowing, a moving median was calculated per channel from the original data (median value over 1 min of original data). Per channel, a fasted ‘moving’ reference value was calculated from the moving median data as the average pressure in the last 5 min before the IV infusion; a fed ‘moving’ reference value was calculated from the moving median data as the average pressure in the last 5 min before the intragastric ND infusion.

Data about IGP in fasted state were presented per minute as the difference between the minimum moving median value in that minute and the fasted ‘moving’ reference value. IGP changes during the intragastric ND infusion were presented per minute as the difference of the minimum moving median value in that minute and the fed ‘moving’ reference value. At this step, the values represent absolute pressure measurements, which are corrected for non‐stomach contractions. The average of absolute IGP values 5 min before PP infusion (fasted baseline) and before ND infusion (fed baseline) was compared between conditions using a repeated one‐way ANOVA.

IGP in the fasted state (period of 30 min before ND infusion) was analyzed as changes from fasted baseline. These delta IGP data were presented as mean ± SEM, and were analyzed using a mixed model analysis. Planned contrast analysis was corrected for multiple testing by Dunnett's test (*p* < 0.05 was considered significant).

The nadir was presented as the difference between the fed baseline IGP values and the lowest absolute IGP value during the meal. The nadir and time to reach nadir were presented as mean ± SEM and compared using repeated one‐way ANOVA (*p* < 0.05 was considered significant). Pearson correlation analysis was used to examine the degree in which the nadir value explains its effect on food tolerance. The values of both variables for PP3 and PP10 were subtracted with the according placebo value (*p* < 0.05 was considered significant).

#### 2.5.2 Volume tolerance, satiation, return of hunger, and prospective food intake

The volume of ND tolerated was represented as mean ± SEM and compared using repeated one‐way ANOVA with Dunnett's test for multiple testing for the three conditions in protocol 1 and two‐tailed, paired t‐test for two conditions in protocol 2 (*p* < 0.05 was considered significant). For the satiation score, satiety, return of hunger, prospective food intake, and symptoms evaluated by VAS, a mixed model analysis was used with condition, time and condition‐by‐time as main output. Additional analysis was performed with Dunnett's test to correct for multiple testing (*p* < 0.05 was considered significant).

#### 2.5.3 Gastric emptying test

The isotope enrichment in the breath samples was measured by isotope ratio mass spectrometry (ABCA, Sercon). Measured delta values were converted to percentage of administered dose of ^13^C excreted per hour assuming a CO_2_ production of 300 mmol*m^−^² of body surface per hour. These data were used to mathematically fit a curve, from which the gastric half‐emptying time (T_1/2_) was subsequently calculated (Ghoos et al., 1993). Data were represented as mean ± SEM and compared with a two‐tailed, paired *t*‐test (*p* < 0.05 was considered significant). Pearson correlation analysis was performed between food tolerance and gastric half‐emptying time (*p* < 0.05 was considered significant).

#### 2.5.4 Human PP assay

PP plasma levels were evaluated on four separate time points as mentioned above. These concentrations serve as indication of the PP plasma accumulation for the different concentrations and had been collected from six volunteers. Mixed model analysis was used to compare between conditions, by time and for a condition‐by‐time effect. Further testing to find an effect between conditions at the same time points was corrected for multiple testing by Dunnett's test (*p* < 0.05 was considered significant).

#### 2.5.5 Statistical power

For this study, we made an estimation for the n‐value based on several earlier studies. Batterham and colleagues showed statistically significant suppressed energy intake by a PP10 dose in 10 individuals (Batterham et al., 2003). A 5 pmol*kg^−1^*min^−1^ PP dose showed a significant reduction of energy intake, in a group of 14 volunteers (Jesudason et al., 2007). Therefore, 12 volunteers for the nutrient tolerance experiment in combination with the IGP measurement was sufficient to demonstrate an effect of the chosen PP doses. Gastric emptying of a solid meal was delayed by infusion of both low PP doses (0.75 and 2.25 pmol*kg^−1^*min^−1^) in a group of eight volunteers. No effect was found on emptying after intake of water, although this was only performed in six volunteers (Schmidt et al., 2005). For the gastric emptying of the nutrient drink, 10 healthy volunteers were recruited.

## 3 RESULTS

### 3.1 Protocol 1: Gastric accommodation and food tolerance

Twelve HVs (age 25 ± 2 y, BMI 23.9 ± 0.7 kg*m^−2^, 6 females) were recruited for this study.

#### 3.1.1 Nutrient tolerance

The maximal tolerated volume was significantly different between conditions (*p* = 0.048; n = 12). The maximal volume of infused ND tended to be increased for PP3, but not for PP10 (*Padj* = 0.07 for PP0 vs. PP3 and *Padj* = 0.14 for PP0 vs. PP10; 886 ± 93 ml for PP0 vs. 1059 ± 124 ml for PP3 and 1025 ± 125 ml for PP10; n = 12).

#### 3.1.2 Intragastric pressure measurements

**FIGURE 1:**
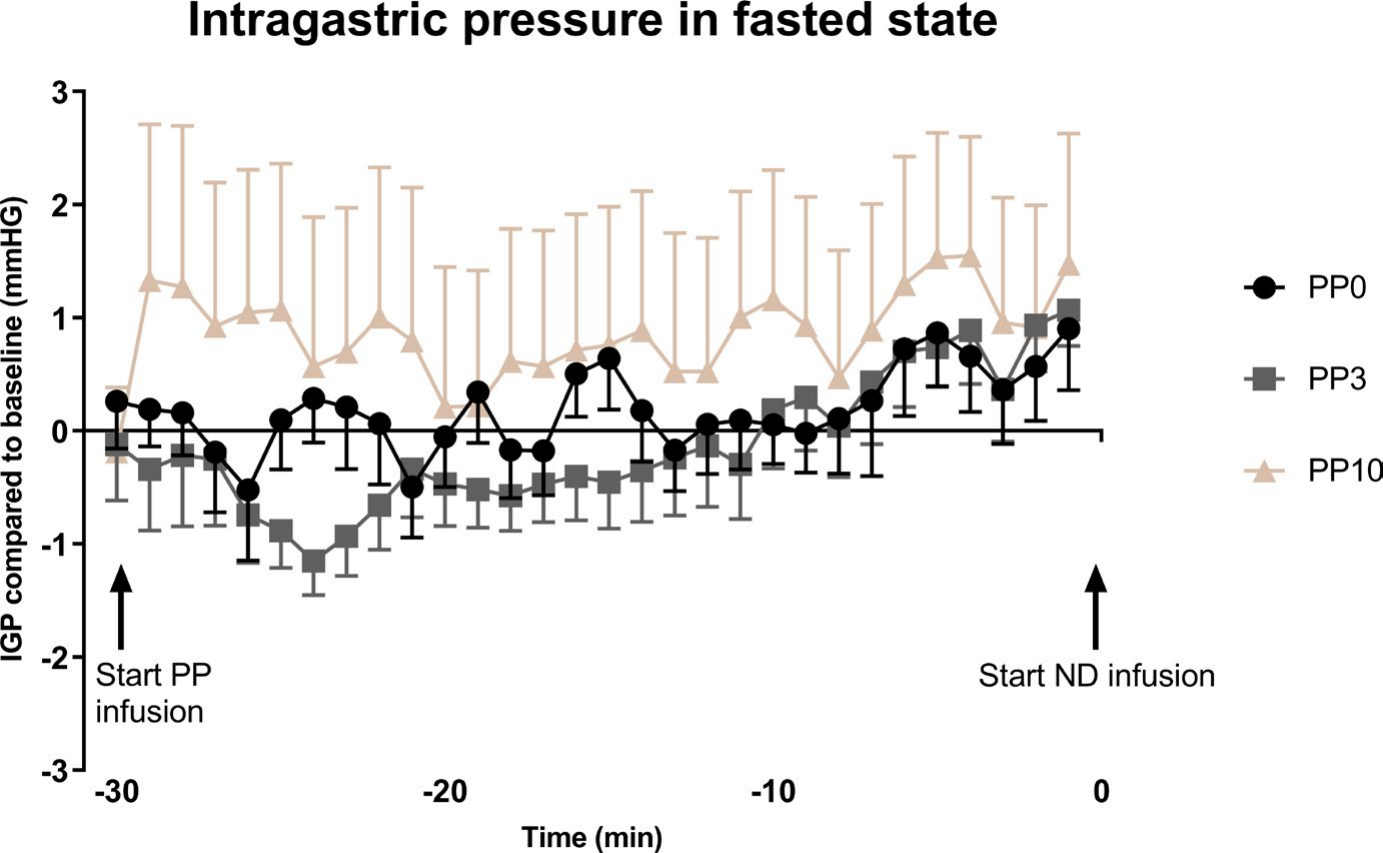
The change of IGP during the first 30 min of treatment in the fasted state. The values are changes from the fasted baseline IGP (average value of 5 min before the treatment). Data are presented as mean ± SEM (n = 12). IGP, intragastric pressure; PP, pancreatic polypeptide; ND, nutrient drink

There was no difference in the fasted IGP baseline (5 min before PP infusion) between conditions (9.8 ± 1.0 mmHg for PP0 vs. 8.8 ± 1.9 mmHg for PP3 vs. 9.6 ± 2.0 mmHg for PP10; *p* = 0.99; n = 6). The delta IGP values during the fasted state (n = 12; Figure 1) were overall not affected by the PP treatments (*p* = 0.37). The delta IGP changed significantly over time (*p* = 0.007) and there was a tendency for a condition‐by‐time effect (*p* = 0.067). Further analysis showed no significant effect for PP3 in comparison with the PP0 (*Padj* = 0.14), but did show a significant delta IGP increase after PP10 administration (*Padj* = 0.013). The last 5‐min values before ND infusion (fed IGP baseline) were not significantly different between conditions (9.3 ± 1.0 mmHg for PP0 vs. 10.7 ± 1.6 mmHg for PP3 vs. 10.6 ± 1.2 mmHg for PP10; *p* = 0.63; n = 12).

**FIGURE 2:**
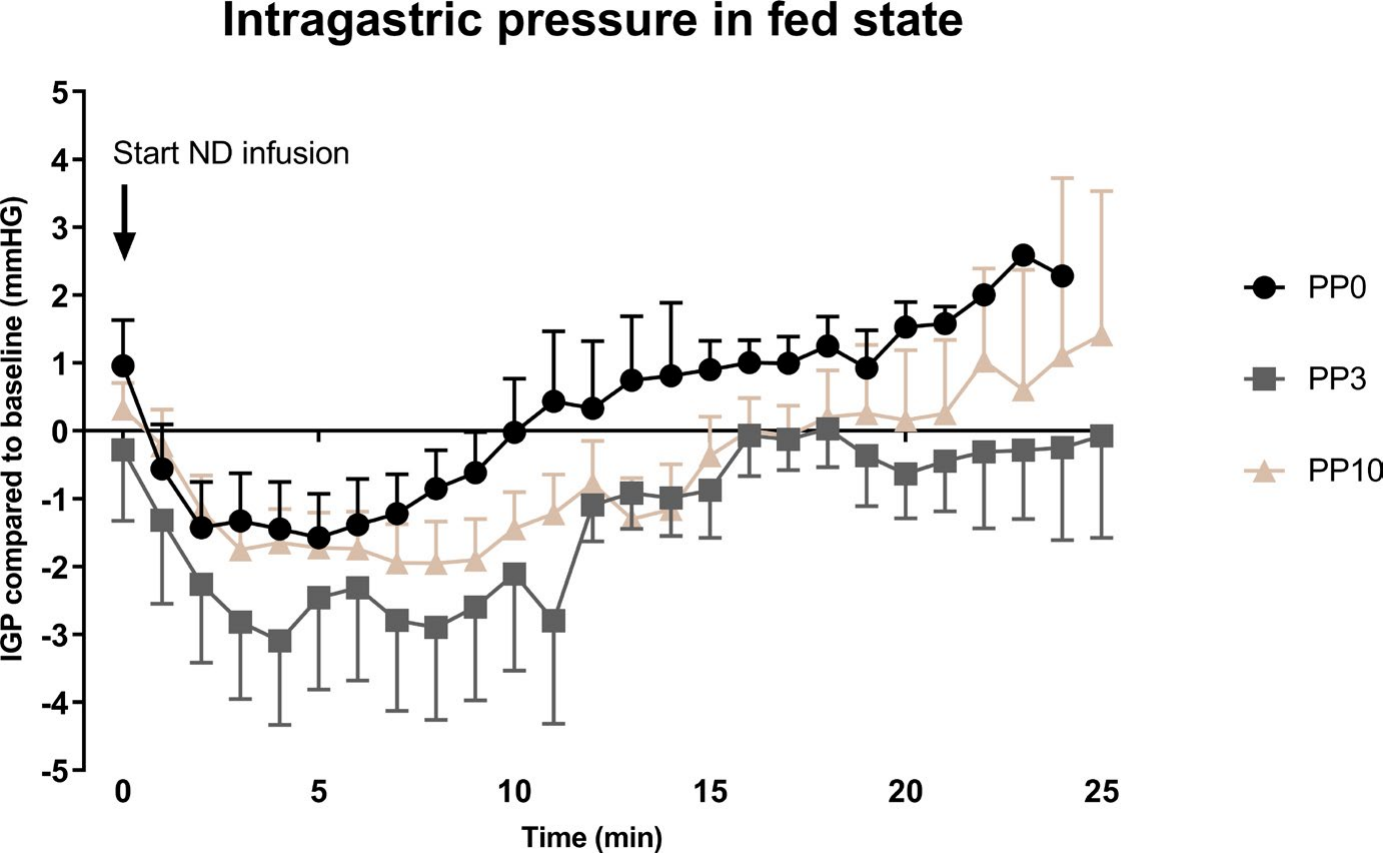
The change of IGP from the start of nutrient drink infusion until 25 min. The values are changes from a baseline pressure (average value of 5 min before nutrient drink infusion). Data are presented as mean ± SEM (n = 12). IGP, intragastric pressure; PP, pancreatic polypeptide; ND, nutrient drink

During intragastric infusion of ND, IGP initially decreased and, thereafter, gradually increased in all the treatment groups (Figure 2). Nadir IGP values were transformed according to the box‐cox method and values were presented as such. No differences were found among PP0, PP3, and PP10 for the nadir IGP values (1.5 ± 0.2 mmHg vs. 1.7 ± 0.3 mmHg vs. 1.6 ± 0.3 mmHg, for PP0, PP3, and PP10, respectively; *p* = 0.82; n = 12) or time to nadir pressure (3.8 ± 0.6 min vs. 6.1 ± 0.8 min vs. 6.5 ± 1.4 min; for PP0, PP3, and PP10, respectively; *p* = 0.32; n = 12). The effect of PP3 and PP10 on the nadir value in comparison with placebo was not correlated with the effect on food tolerance (R² = 0.001, *p* = 0.81, and R² = 0.04, *p* = 0.52, respectively).

#### 3.1.3 Human PP assay

**FIGURE 3:**
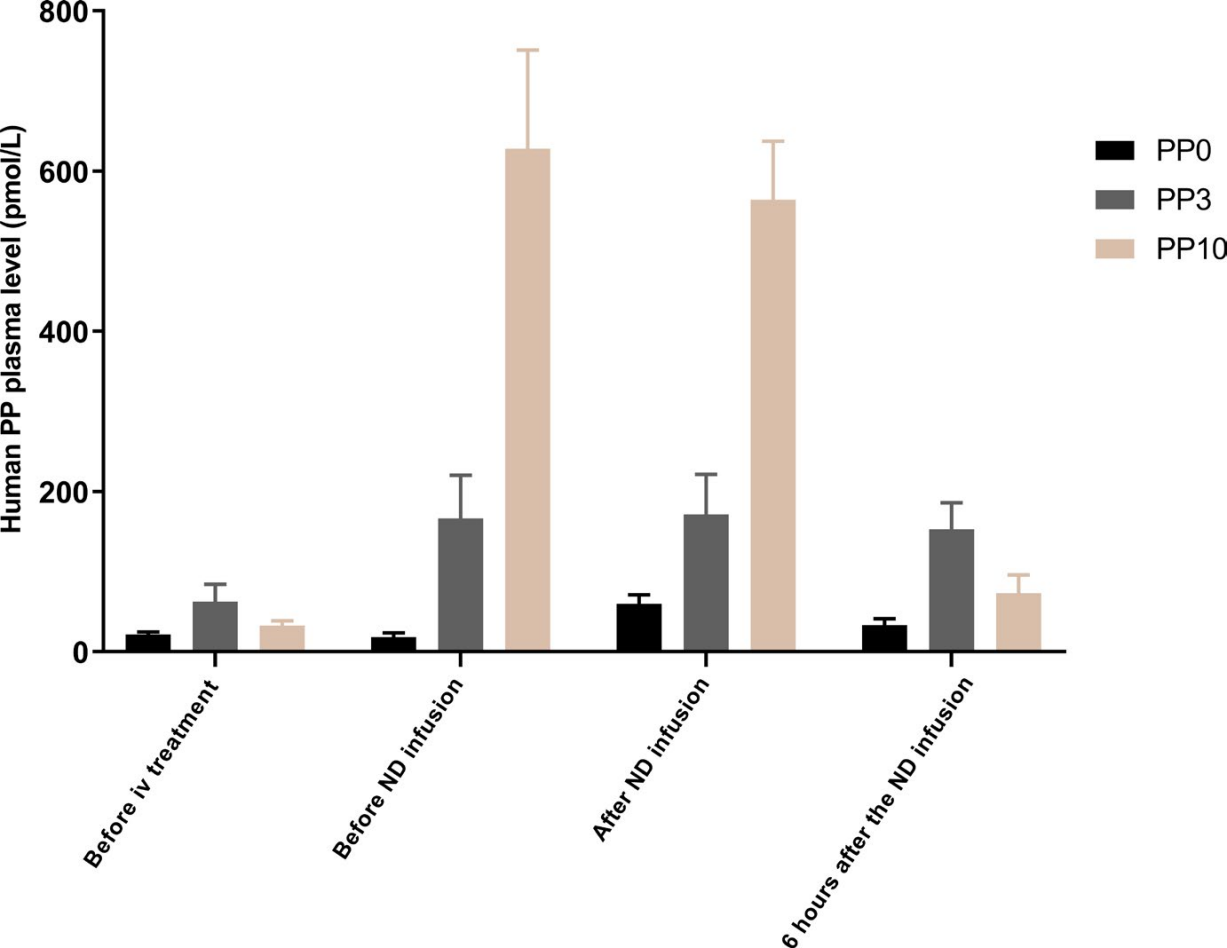
Human PP plasma concentrations before treatment of placebo (PP0) and 3 pmol*kg^−1^*min^−1^ PP (PP3) and 10 pmol*kg^−1^*min^−1^ PP (PP10) 5 min before and immediately after intragastric ND infusion and 6 h after the ND infusion. Data are represented as mean ± SEM (n = 6) and *p* < 0.05 was considered significant. PP, pancreatic polypeptide

Human PP plasma concentrations are described in Table S1 and are shown in Figure 3. For the analysis, these data were logarithmically transformed and are presented as such in this section. During the fasting state, prior to the start of the infusions, log PP plasma values were 3.0 ± 0.17 pmol/L for PP0, 4.0 ± 0.3 pmol/L for PP3, and 3.4 ± 0.2 pmol/L for PP10 treatment, respectively (*Padj* = 0.34; n = 6).

Before the intragastric ND infusion, log PP concentrations were significantly higher after PP3 and PP10 infusion than in the PP0 group (2.7 ± 0.3 pmol/L after PP0 vs. 4.6 ± 0.5 pmol/L after PP3 vs. 6.3 ± 0.2 pmol/L after PP10, *Padj* < 0.0001; n = 6). After the intragastric ND infusion, log PP concentrations were significantly higher after PP3 and PP10 in comparison with the placebo (4.0 ± 0.3 pmol/L after PP0 vs. 4.9 ± 0.4 pmol/L after PP3 vs. 6.3 ± 0.1 pmol/L after PP10, *Padj* < 0.0001; n = 6). Significant differences in log PP plasma concentrations were found 6 hours after the ND administration amongst the three groups (3.4 ± 0.2 pmol/L after PP0 vs. 4.9 ± 0.3 pmol/L after PP3 vs. 4.1 ± 0.2 pmol/L after PP10; *Padj* = 0.046; n = 6).

### 3.2 Protocol 2: Gastric emptying and food tolerance

Ten HVs (age 23 ± 1 y, BMI 22.9 ± 0.6 kg*m^−2^, 6 females) were recruited for this study.

#### 3.2.1 Nutrient tolerance

The maximal tolerated volume was significantly different between placebo and PP10 (802 ± 119 ml vs. 1089 ± 128 ml, for PP0 and PP10, respectively; *p* = 0.016; n = 10).

#### 3.2.2 Gastric emptying rate

Gastric emptying of the infused nutrient meal was similar between PP0 and PP10 treatments (*p* = 0.49; n = 10). The gastric half‐emptying time was 281 ± 52 min in PP0 and 249 ± 37 min in PP10. A significant correlation was found between the volume of ND ingested and gastric half‐emptying time in PP0 (*p* = 0.013; R² = 0.56), while this correlation was lost in PP10 treatment (*p* = 0.32; R² = 0.12).

#### 3.2.3 Satiety, return of hunger, and prospective food intake

**FIGURE 4:**
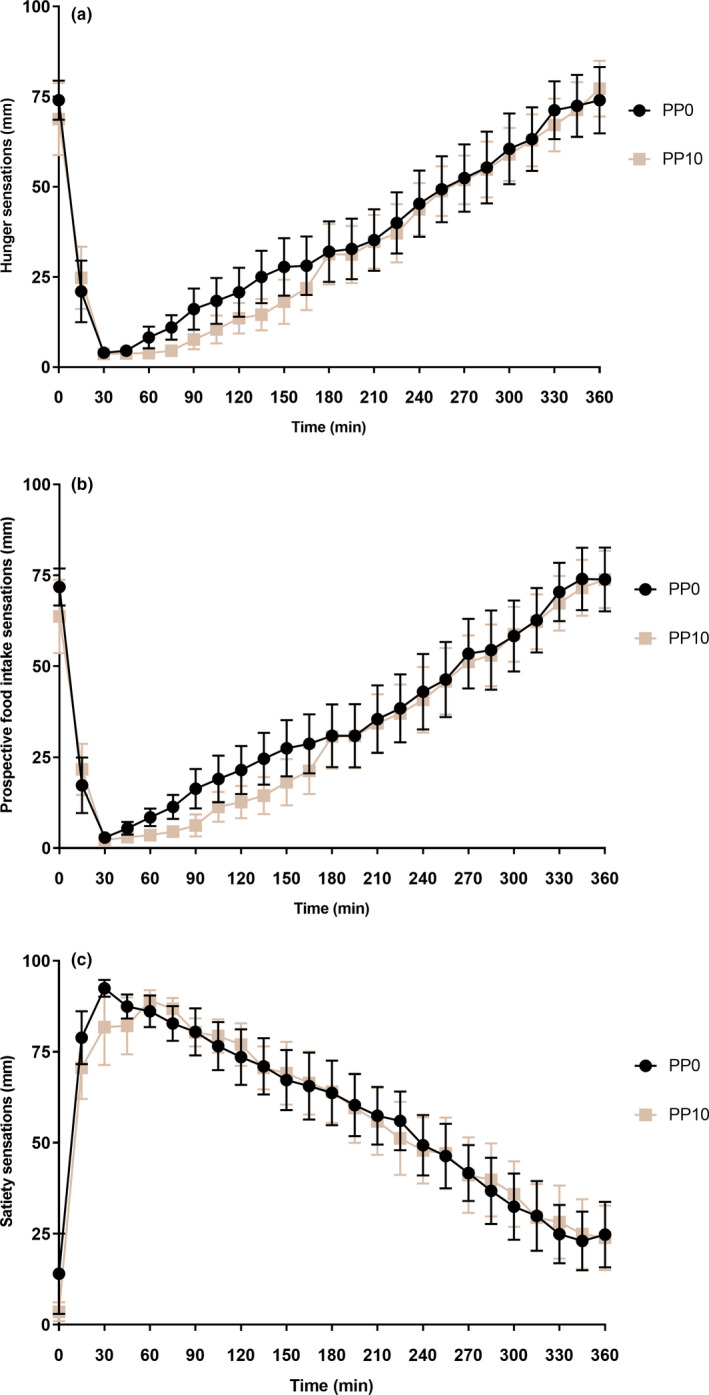
Appetite‐related sensations scored during the gastric emptying experiment on visual analogue scale scores (0–100 mm) between placebo (PP0) and 10 pmol*kg^−1^*min^−1^ PP (PP10). T_0_ represents the start of the intragastric nutrient drink infusion. Data are represented as mean ± SEM (n = 9), and an effect is considered significant if *p* < 0.05. (a) Hunger sensations; (b) Prospective food intake sensations and (c) Satiety sensations. PP, pancreatic polypeptide

Data for appetite sensations were missing for one volunteer. During the gastric emptying study, hunger sensations were not overall affected by PP doses (*p* = 0.55; n = 9). The sensations did decrease significantly after the ND (*p* < 0.0001; n = 9), although the change over time was not affected by the condition (*p* = 0.67; n = 9; Figure 4a). Prospective food intake scores followed a similar pattern as hunger; the condition had no significant effect (*p* = 0.47; n = 9). Prospective food intake scores decreased after nutrient infusion (*p* < 0.0001; n = 9), and this time difference was affected by the PP10 treatment (*p* = 0.08; n = 9; Figure 4b). Satiety sensations were not affected by condition (*p* = 0.92; n = 9), in contrast to hunger these feelings increased over time (*p* < 0.0001; n = 9), but the change over time was not different between PP0 and PP10 (*p* = 0.76; n = 9; Figure 4c).

## 4 DISCUSSION

In this exploratory study, we examined the effects of an intravenous infusion of 3 pmol/kg*min (PP3) and 10 pmol/kg*min (PP10) PP in comparison with placebo (PP0) on nutrient volume tolerance, fasted and fed intragastric pressure, gastric emptying, and food‐related sensations in normal‐weight healthy adults. Based on our findings, we suggest that a supraphysiological dose of PP increases meal volume tolerance, but this is not mediated by an effect on gastric accommodation or gastric emptying.

PP is a neuropeptide Y family member, which is released from pancreatic F cells. Neuropeptide Y (NPY) and peptide YY (PYY) are its relatives, and together they exhibit a governing role on food intake and homeostatic functions after binding one of the Y1–Y5 receptors. An important role for PP in the modulation of food intake has been suggested earlier, as its administration affects food intake in animals and altered secretion of PP is associated with eating disorders in humans. PP has been predominantly described as anorexigenic in humans, although findings in animals are inconsistent. An intracerebroventricular PP injection stimulates food intake in a dose dependently fashion in mice, whereas intraperitoneal injection dose dependently decreases food intake (Asakawa et al., 1999; Clark et al., 1984). A similar effect is observed in dogs, where central PP administration stimulates feeding (Inui et al., 1991), while intraperitoneal injections hamper eating motivation (Akerberg et al., 2010). It seems plausible that PP controls food intake by a central and peripheral site‐specific activity. PP primarily binds Y4 receptor and secondarily the Y5 receptor (Parker et al., 2002). Y4 mRNA is densely expressed in the periphery and parts of the mouse brain, e.g., the area postrema (AP), nucleus tractus solitarius (NTS), dorsal medial ventricle (DMV), and lateral hypothalamus (Campbell et al., 2003; Larsen & Kristensen, 1997; Parker & Herzog, 1999), while Y5 mRNA is more abundant in the lateral hypothalamus (Parker & Herzog, 1999). Although PP has limited access through the blood–brain barrier, it is possible that it reaches these brain regions via the fenestrated AP (Whitcomb et al., 1990). NPY stimulates food intake after activating hunger‐signaling neurons via Y5 receptor in the arcuate nucleus (Naveilhan et al., 1998) and blocks melanocortin signaling to POMC cells (Lam et al., 2017). We suggest that the supraphysiological PP concentrations reached in this study were able to bind Y5 brain receptors, therefore outclassing the anorexigenic effect. Our results considering food intake are in disagreement with the findings of Batterham et al. with IV PP infusion. A first possible explanation is our higher PP10 accumulation in the plasma, which is almost 10 times higher in comparison with the PP increase after ND infusion (physiological fed state level). Secondly, Batterham et al. offered a solid meal to their volunteers, while we infused a liquid meal until fully satisfaction. This subsequently means that due to this experimental design of a nasogastric infused meal, the effect of the cephalic phase is bypassed. Lastly, the PP infusion occurred at different time points in the study by Batterham et al. (Batterham et al., 2003).

Gastric tone reflects activity of the excitatory and inhibitory motor neurons activity, which innervate the gastric circular and the longitudinal muscle layers (Azpiroz et al., 2014). The major excitatory neurotransmitter and primary modulator of the gastric tone is acetylcholine, as demonstrated by several studies in both in animal models and humans, showing that the administration of the muscarinic receptor antagonist, atropine, decreases the muscular tone in the stomach (Cuomo et al., 2006; Lidums et al., 2000; Paterson et al., 2000; Rao et al., 2005). It has been demonstrated that PP acts in the gastrointestinal tract by inhibiting cholinergic pathways; studies in guinea pigs showed that PP inhibits the occurrence of cholinergic contraction in intestinal strips (Holzer et al., 1986) and inhibits the release of acetylcholine from myenteric neurons (Schemann & Tamura, 1992; Takahashi et al., 1992). Our data show that a PP10 dose increases the gastric tone in comparison with the other conditions, although this difference disappeared 30 min after the start of PP infusion. As we observed no difference in average IGP 5 min before ND infusion in the fasted state, we conclude that a potential change in fasted IGP had no influence on GA or food intake.

Here, we measured gastric accommodation as the change in intragastric pressure (IGP) during intragastric liquid meal infusion (Janssen, Vanden Berghe, et al., 2011; Scarpellini et al., 2021). Gastric accommodation is a vago‐vagally mediated reflex that occurs in the stomach during ingestion of a meal in order to provide a reservoir for ingested food and consists of a reduction in gastric tone and an increase in compliance in response to food intake, allowing for an increased fundic volume without a rise of IGP. In this study, we observe no inhibitory effect of PP on IGP during a nutrient drink challenge as was observed in the mouse model (Verschueren et al., 2014) or according what could be expected with the increased food intake.

Our results show that the PP10 dose also does not affect the gastric emptying rate. Studies have shown that intraperitoneal injection of PP stimulates gastric emptying in experimental animals (Nakajima et al., 1994), but in general data on gastric emptying rate are discordant, showing a delay, stimulation, or lack of a PP effect (Janssen, Vanden Berghe, et al., 2011; Kojima et al., 2007; Nakajima et al., 1994; Schmidt et al., 2005). Okumura et al (1994) have shown that intraperitoneal injection of PP suppresses gastric emptying in experimental animals, and it seems that the dose of exogenous PP required for inhibition of gastric emptying is comparable to the dose required for suppression of food intake; on the other hand, the intracisternal administration of PP, that may bind the receptor localized in the dorsal vagal complex, accelerated gastric emptying rate in rats and this effect can be induced when PP is centrally administered and is potentially also responsible, at least in part, for the increased food intake (Okumura et al., 1994). Individual volunteer data of half‐emptying time often show a biphasic curve, with a secondarily occurring delay. Volunteers drank, on average, 895.3 ml for placebo and 1089 ml for PP10, which are significant amounts. It is plausible that, due to the experimental design of food tolerance, the caloric intake affected the gastric emptying, for instance, by feedback inhibition from the small bowel. In this experimental setting, the increased volume of food tolerance is not explained by changes in gastric emptying.

There are a few limitations in this study. First, plasma for the PP assay was only collected from six participants to confirm the delivery in the blood stream, and to estimate the PP concentration ranges by the different doses. Second, the nutrient tolerance test bypasses the cephalic phase and is developed to elucidate the functioning of the gastric motor mechanism (Scarpellini et al., 2021). It is true that the taste of mixed meals stimulates PP secretion into the plasma; however, this moderate release (±30 pg/ml) (Teff, 2010) is negligible compared to the PP concentrations after PP3 or PP10 administration.

In conclusion, the present study suggests that the exogenous administration of PP in HVs increases tolerance of an intragastrically infused nutrient meal. No effect on gastric accommodation or gastric emptying is observed, and postprandial food‐related sensations are not affected.

## CONFLICT OF INTEREST

None of the authors have a conflict of interest to declare.

## AUTHOR CONTRIBUTIONS

The authors’ responsibilities were as followed – AR, PJ, and JT designed research; AR conducted the research; WV and FC analyzed data or performed statistical analysis; WV, FC, PJ, and JT wrote the paper; all authors had primary responsibility for final content.

## Supporting information



Table S1

## References

[phy215002-bib-0001] Akerberg, H., Meyerson, B., Sallander, M., Lagerstedt, A. S., Hedhammar, A., & Larhammar, D. (2010). Peripheral administration of pancreatic polypeptide inhibits components of food‐intake behavior in dogs. Peptides, 31(6), 1055–1061. 10.1016/j.peptides.2010.03.019 20338207

[phy215002-bib-0002] Asakawa, A., Inui, A., Ueno, N., Fujimiya, M., Fujino, M. A., & Kasuga, M. (1999). Mouse pancreatic polypeptide modulates food intake, while not influencing anxiety in mice. Peptides, 20(12), 1445–1448. 10.1016/S0196-9781(99)00155-2 10698120

[phy215002-bib-0003] Azpiroz, F., Feinle‐Bisset, C., Grundy, D., & Tack, J. (2014). Gastric sensitivity and reflexes: basic mechanisms underlying clinical problems. Journal of Gastroenterology, 49(2), 206–218. 10.1007/s00535-013-0917-8 24306100

[phy215002-bib-0004] Batterham, R. L., Le Roux, C. W., Cohen, M. A., Park, A. J., Ellis, S. M., Patterson, M., Frost, G. S., Ghatei, M. A., & Bloom, S. R. (2003). Pancreatic polypeptide reduces appetite and food intake in humans. The Journal of Clinical Endocrinology and Metabolism, 88(8), 3989–3992. 10.1210/jc.2003-030630 12915697

[phy215002-bib-0005] Berntson, G. G., Zipf, W. B., O'Dorisio, T. M., Hoffman, J. A., & Chance, R. E. (1993). Pancreatic polypeptide infusions reduce food intake in Prader‐Willi syndrome. Peptides, 14(3), 497–503. 10.1016/0196-9781(93)90138-7 8332550

[phy215002-bib-0006] Braden, B., Adams, S., Duan, L.‐P., Orth, K.‐H., Maul, F.‐D., Lembcke, B., Hör, G., & Caspary, W. F. (1995). The [13C]acetate breath test accurately reflects gastric emptying of liquids in both liquid and semisolid test meals. Gastroenterology, 108(4), 1048–1055. 10.1016/0016-5085(95)90202-3 7698571

[phy215002-bib-0007] Campbell, R. E., Smith, M. S., Allen, S. E., Grayson, B. E., Ffrench‐Mullen, J. M., & Grove, K. L. (2003). Orexin neurons express a functional pancreatic polypeptide Y4 receptor. The Journal of Neuroscience, 23(4), 1487–1497. 10.1523/JNEUROSCI.23-04-01487.2003 12598637 PMC6742240

[phy215002-bib-0008] Clark, J. T., Kalra, P. S., Crowley, W. R., & Kalra, S. P. (1984). Neuropeptide Y and human pancreatic polypeptide stimulate feeding behavior in rats. Endocrinology, 115(1), 427–429. 10.1210/endo-115-1-427 6547387

[phy215002-bib-0009] Cuomo, R., Vandaele, P., Coulie, B., Peeters, T., Depoortere, I., Janssens, J., & Tack, J. (2006). Influence of motilin on gastric fundus tone and on meal‐induced satiety in man: Role of cholinergic pathways. The American Journal of Gastroenterology, 101(4), 804–811. 10.1111/j.1572-0241.2005.00339.x 16635226

[phy215002-bib-0010] Ekblad, E., & Sundler, F. (2002). Distribution of pancreatic polypeptide and peptide YY. Peptides, 23(2), 251–261. 10.1016/S0196-9781(01)00601-5 11825640

[phy215002-bib-0011] Fujimoto, S., Inui, A., Kiyota, N., Seki, W., Koide, K., Takamiya, S., Uemoto, M., Nakajima, Y., Baba, S., & Kasuga, M. (1997). Increased cholecystokinin and pancreatic polypeptide responses to a fat‐rich meal in patients with restrictive but not bulimic anorexia nervosa. Biological Psychiatry, 41(10), 1068–1070. 10.1016/S0006-3223(97)00044-9 9129788

[phy215002-bib-0012] Ghoos, Y. F., Maes, B. D., Geypens, B. J., Mys, G., Hiele, M. I., Rutgeerts, P. J., & Vantrappen, G. (1993). Measurement of gastric emptying rate of solids by means of a carbon‐labeled octanoic acid breath test. Gastroenterology, 104(6), 1640–1647. 10.1016/0016-5085(93)90640-X 8500721

[phy215002-bib-0013] Holzer, P., Bartho, L., Lippe, I. T., Petritsch, W., & Leb, G. (1986). Effect of pancreatic polypeptide on the motility of the guinea‐pig small intestine in vitro. Regulatory Peptides, 16(3‐4), 305–314. 10.1016/0167-0115(86)90030-3 3562903

[phy215002-bib-0014] Inui, A., Okita, M., Nakajima, M., Inoue, T., Sakatani, N., Oya, M., Morioka, H., Okimura, Y., Chihara, K., & Baba, S. (1991). Neuropeptide regulation of feeding in dogs. American Journal of Physiology‐Regulatory, Integrative and Comparative Physiology, 261(3), R588–R594. 10.1152/ajpregu.1991.261.3.R588 1716066

[phy215002-bib-0015] Janssen, P., Vanden Berghe, P., Verschueren, S., Lehmann, A., Depoortere, I., & Tack, J. (2011). Review article: the role of gastric motility in the control of food intake. Alimentary Pharmacology and Therapeutics, 33(8), 880–894. 10.1111/j.1365-2036.2011.04609.x. 21342212

[phy215002-bib-0016] Janssen, P., Verschueren, S., Ly, H. G., Vos, R., Van Oudenhove, L., & Tack, J. (2011). Intragastric pressure during food intake: a physiological and minimally invasive method to assess gastric accommodation. Neurogastroenterology and Motility, 23(4), 316–322, e153–4. 21299720 10.1111/j.1365-2982.2011.01676.x

[phy215002-bib-0017] Jesudason, D. R., Monteiro, M. P., McGowan, B. M. C., Neary, N. M., Park, A. J., Philippou, E., Small, C. J., Frost, G. S., Ghatei, M. A., & Bloom, S. R. (2007). Low‐dose pancreatic polypeptide inhibits food intake in man. British Journal of Nutrition, 97(3), 426–429. 10.1017/S0007114507336799 17313701

[phy215002-bib-0018] Kojima, S., Ueno, N., Asakawa, A., Sagiyama, K., Naruo, T., Mizuno, S., & Inui, A. (2007). A role for pancreatic polypeptide in feeding and body weight regulation. Peptides, 28(2), 459–463. 10.1016/j.peptides.2006.09.024 17207558

[phy215002-bib-0019] Lam, B. Y. H., Cimino, I., Polex‐Wolf, J., Nicole Kohnke, S., Rimmington, D., Iyemere, V., Heeley, N., Cossetti, C., Schulte, R., Saraiva, L. R., Logan, D. W., Blouet, C., O'Rahilly, S., Coll, A. P., & Yeo, G. S. H. (2017). Heterogeneity of hypothalamic pro‐opiomelanocortin‐expressing neurons revealed by single‐cell RNA sequencing. Molecular Metabolism, 6(5), 383–392. 10.1016/j.molmet.2017.02.007 28462073 PMC5404100

[phy215002-bib-0020] Larhammar, D. (1996). Structural diversity of receptors for neuropeptide Y, peptide YY and pancreatic polypeptide. Regulatory Peptides, 65(3), 165–174. 10.1016/0167-0115(96)00110-3 8897639

[phy215002-bib-0021] Larsen, P. J., & Kristensen, P. (1997). The neuropeptide Y (Y4) receptor is highly expressed in neurones of the rat dorsal vagal complex. Molecular Brain Research, 48(1), 1–6. 10.1016/S0169-328X(97)00069-7 9379829

[phy215002-bib-0022] Lidums, I., Hebbard, G. S., & Holloway, R. H. (2000). Effect of atropine on proximal gastric motor and sensory function in normal subjects. Gut, 47(1), 30–36. 10.1136/gut.47.1.30 10861261 PMC1727959

[phy215002-bib-0023] Lieverse, R. J., Masclee, A. A., Jansen, J. B., & Lamers, C. B. (1994). Plasma cholecystokinin and pancreatic polypeptide secretion in response to bombesin, meal ingestion and modified sham feeding in lean and obese persons. International Journal of Obesity and Related Metabolic Disorders, 18(2), 123–127. 8148926

[phy215002-bib-0024] Nakajima, M., Inui, A., Teranishi, A., Miura, M., Hirosue, Y., Okita, M., Himori, N., Baba, S., & Kasuga, M. (1994). Effects of pancreatic polypeptide family peptides on feeding and learning behavior in mice. Journal of Pharmacology and Experimental Therapeutics, 268(2), 1010–1014. 8113957

[phy215002-bib-0025] Naveilhan, P., Neveu, I., Arenas, E., & Ernfors, P. (1998). Complementary and overlapping expression of Y1, Y2 and Y5 receptors in the developing and adult mouse nervous system. Neuroscience, 87(1), 289–302. 10.1016/S0306-4522(98)00141-9 9722158

[phy215002-bib-0026] Okumura, T., Pappas, T. N., & Taylor, I. L. (1994). Intracisternal injection of pancreatic polypeptide stimulates gastric emptying in rats. Neuroscience Letters, 178(1), 167–170. 10.1016/0304-3940(94)90316-6 7816328

[phy215002-bib-0027] Parker, M. S., Lundell, I., & Parker, S. L. (2002). Pancreatic polypeptide receptors: Affinity, sodium sensitivity and stability of agonist binding. Peptides, 23(2), 291–303. 11825644 10.1016/s0196-9781(01)00610-6

[phy215002-bib-0028] Parker, R. M., & Herzog, H. (1999). Regional distribution of Y‐receptor subtype mRNAs in rat brain. European Journal of Neuroscience, 11(4), 1431–1448. 10.1046/j.1460-9568.1999.00553.x 10103138

[phy215002-bib-0029] Paterson, C. A., Anvari, M., Tougas, G., & Huizinga, J. D. (2000). Nitrergic and cholinergic vagal pathways involved in the regulation of canine proximal gastric tone: an in vivo study. Neurogastroenterology and Motility, 12(4), 301–306. 10.1046/j.1365-2982.2000.00209.x 10886672

[phy215002-bib-0030] Rao, S. S., Kumar, A., Harris, B., Brown, B., & Schulze, K. S. (2005). Investigation of fundo‐antral reflex in human beings. World Journal of Gastroenterology, 11(42), 6676– 10.3748/wjg.v11.i42.6676 16425364 PMC4355764

[phy215002-bib-0031] Scarpellini, E., Van den Houte, K., Schol, J., Huang, I.‐H., Colomier, E., Carbone, F., & Tack, J. (2021). Nutrient drinking test as biomarker in functional dyspepsia. American Journal of Gastroenterology, 116(7), 1387–1395. 10.14309/ajg.0000000000001242 33941747

[phy215002-bib-0032] Schemann, M., & Tamura, K. (1992). Presynaptic inhibitory effects of the peptides NPY, PYY and PP on nicotinic EPSPs in guinea‐pig gastric myenteric neurones. The Journal of Physiology, 451(1), 79–89. 10.1113/jphysiol.1992.sp019154 1403832 PMC1176151

[phy215002-bib-0033] Schmidt, P. T., Näslund, E., Grybäck, P., Jacobsson, H., Holst, J. J., Hilsted, L., & Hellström, P. M. (2005). A role for pancreatic polypeptide in the regulation of gastric emptying and short‐term metabolic control. The Journal of Clinical Endocrinology and Metabolism, 90(9), 5241–5246. 10.1210/jc.2004-2089 15998783

[phy215002-bib-0034] Takahashi, T., Yamamura, T., & Utsunomiya, J. (1992). Human pancreatic polypeptide, neuropeptide Y and peptide YY reduce the contractile motility by depressing the release of acetylcholine from the myenteric plexus of the guinea pig ileum. Gastroenterologia Japonica, 27(3), 327–333. 10.1007/BF02777750 1624076

[phy215002-bib-0035] Tatemoto, K., Carlquist, M., & Mutt, V. (1982). Neuropeptide Y—a novel brain peptide with structural similarities to peptide YY and pancreatic polypeptide. Nature, 296(5858), 659–660. 10.1038/296659a0 6896083

[phy215002-bib-0036] Teff, K. L. (2010). Cephalic phase pancreatic polypeptide responses to liquid and solid stimuli in humans. Physiology and Behavior, 99(3), 317–323. 10.1016/j.physbeh.2009.11.009 19944113 PMC2834473

[phy215002-bib-0037] Verschueren, S., Janssen, P., Van Oudenhove, L., Hultin, L., & Tack, J. (2014). Effect of pancreatic polypeptide on gastric accommodation and gastric emptying in conscious rats. American Journal of Physiology. Gastrointestinal and Liver Physiology, 307(1), G122–G128. 10.1152/ajpgi.00043.2014. 24742985

[phy215002-bib-0038] Whitcomb, D. C., Taylor, I. L., & Vigna, S. R. (1990). Characterization of saturable binding sites for circulating pancreatic polypeptide in rat brain. American Journal of Physiology‐Gastrointestinal and Liver Physiology, 259(4), G687–G691. 10.1152/ajpgi.1990.259.4.G687 2221079

[phy215002-bib-0039] Zipf, W. B., O'Dorisio, T. M., Cataland, S., & Sotos, J. (1981). Blunted pancreatic polypeptide responses in children with obesity of Prader‐Willi syndrome. The Journal of Clinical Endocrinology and Metabolism, 52(6), 1264–1266. 10.1210/jcem-52-6-1264 7014602

