# Supplementary material for: Supraphysiological effects of pancreatic polypeptide on gastric motor function and nutrient tolerance in humans
Source: Physiol Rep. 2021 Aug 26;9(17):e15002. doi: 10.14814/phy2.15002 (PMC8387790; doi:10.14814/phy2.15002)
Supplement: Supplementary file 1 — Table S1 [file PHY2-9-e15002-s001.docx]

Supplement table 1. The non-transformed pancreatic polypeptide concentrations for three conditions: PP0; PP3 and PP10. Samples were collected on four different time points: 5 minutes before the pancreatic polypeptide infusion (time point 1); 5 minutes before the intragastric nutrient drink infusion (time point 2); at the end of the nutrient drink infusion (time point 3) and 6 hours after the meal (time point 4). Values are presented as the mean±SEM of six volunteers.

| Condition | Time point | Mean±SEM concentration (pmol/L) |
| --- | --- | --- |
| PP0 | 1 | 21.2 ± 3.6 |
|  | 2 | 18.3 ± 5.4 |
|  | 3 | 59.9 ± 11.3 |
|  | 4 | 32.9 ± 8.5 |
| PP3 | 1 | 62.4 ± 21.9 |
|  | 2 | 166.6 ± 53.6 |
|  | 3 | 171.5 ± 49.9 |
|  | 4 | 152.7 ± 33.3 |
| PP10 | 1 | 32.6 ± 6.0 |
|  | 2 | 627.8 ± 123.5 |
|  | 3 | 564.1 ± 73.3 |
|  | 4 | 72.8 ± 23.3 |
